# A neutral ceramidase, *NlnCDase*, is involved in the stress responses of brown planthopper, *Nilaparvata lugens* (Stål)

**DOI:** 10.1038/s41598-018-19219-y

**Published:** 2018-01-18

**Authors:** Xiao-Xiao Shi, Yuan-Jie Huang, Mahfuj-Ara Begum, Mu-Fei Zhu, Fei-Qiang Li, Min-Jing Zhang, Wen-Wu Zhou, Cungui Mao, Zeng-Rong Zhu

**Affiliations:** 1 State Key Laboratory of Rice Biology, Key Laboratory of Molecular Biology of Crop Pathogens and Insects, Ministry of Agriculture, and Institute of Insect Sciences, Zhejiang University, Hangzhou, Zhejiang 310058 China; 2 Department of Medicine and Stony Brook Cancer Center, The State University of New York at Stony Brook, Stony Brook, New York, 11794 USA

## Abstract

Ceramidases (CDases) are vital enzymes involved in the biosynthesis of sphingolipids, which are essential components of eukaryotic membranes. The function of these enzymes in insects, however, is poorly understood. We identified a neutral ceramidase (NlnCDase) from the brown planthopper, *Nilaparvata lugens*, one of the most destructive hemipteran pests of rice. The C12-ceramide was the most preferred substrate for the NlnCDase enzyme. The activity of the NlnCDase enzyme was highest in the neutral-pH range (pH 6.0). It was inhibited by EGTA, Cs^+^ and Fe^2+^, while stimulated by EDTA and Ca^2+^. Moreover, the NlnCDase has higher transcript level and activity in adults than in eggs and nymphs, and in the reproductive organs (ovaries and spermaries) than in other tissues (i.e. heads, thorax, legs, midguts), which suggested that the NlnCDase might be elevated to mediate developmental process. In addition, transcripts and activity of the NlnCDase were up-regulated under abiotic stresses including starvation, abnormal temperature, and insecticides, and biotic stress of resistant rice varieties. Knocking down *NlnCDase* by RNA interference increased female survival under starvation and temperature stresses, suggesting that NlnCDase might be involved in the stress response in *N*. *lugens*.

## Introduction

Sphingolipids are essential, ubiquitous, and bioactive lipid components of eukaryotic membranes. The metabolites of sphingolipid biosynthesis pathway, such as ceramides, sphingosines, and sphingosine-1-phosphates, can regulate numerous cellular processes. Ceramides are the precursors of other complex sphingolipids, and plentiful studies proved that ceramides can mediate cell cycle arrest^1^, cell differentiation^2,3^, and apoptosis^4^. Ceramide can be metabolized into sphingosine, the first metabolite of sphingolipid pathway, which can inhibits the activity of several protein kinases including protein kinase C^5,6^. Sphingosines are later converted into sphingosine-1- phosphates which regulate cell proliferation^7^ and prevent programmed cell death^8^. A variety of stresses, including heat shock, ultraviolet radiation, injury, and infections, are found to modify the level of sphingolipids through affecting their biosynthetic enzymes^9^. Therefore, the biochemical characterization of these enzymes will be helpful for uncovering the roles of their lipid products in stress responses.

Ceramidases (CDases, EC 3.5.1.23) are a group of enzymes whose main function is to hydrolyze the *N*-acyl linkage of ceramides and yield free fatty acids and sphingosines^10^. Sphingosines are further phosphorylated into sphingosine-1-phosphates via the enzyme sphingosine kinase. Due to the fact that intracellular sphingosines are the exclusive catabolic products of intracellular sphingolipids, the hydrolysis of ceramides by CDase is suggested as the rate-limiting step in keeping the balance between ceramide and sphingosine^11^. Moreover, the dynamic balance between the intracellular ceramide and sphingosine-1-phosphate levels is also tightly regulated by the CDase, which determines the survival of the cell^12,13^. The CDase are normally classified into three subfamilies: acid CDases (aCDase)^14,15^, neutral CDases (nCDase)^16,17^, and alkaline CDases (alCDase)^18,19^, based on their catalytic pH optimum, primary structure and localization. In recent years, nCDase have been cloned from mouse^16^, rat^20^, zebra fish^21^, human^22^, camel^23^, wheat^24^, *Arabidopsis*^25^, *Aspergillus oryzae*^26^ and slime mould^27^.

In mammals, the reduction of aCDase in mouse caused some histopathological changes including the early embryonic lethality and the accumulation of ceramide in kidney^28^. And the mice lacking nCDase showed a marked leukocytosis, circulating neutrophils and lymphocytes, which caused by an unexpected increase of sphingosine-1-phosphate^13^. The knockdown of nCDase in zebra fish also led to a decrease in the number of embryos and an increase in apoptotic cells^21^. In *Arabidopsis*, the nCDase is proved to be responsible for plant’s sensitivity to the oxidative stress^25^.

The function of CDases in insects is poorly understood, whilst these enzymes have been characterized in three species: the fruit fly (*Drosophila melanoganster*)^17^, the red flour beetle^29^, and the small brown planthopper (*Laodelphax striatellus*)^30^. Inactivation of alCDase not only prolonged the development time and lifespan of *D*. *melanogaster*, but also improved the anti-oxidative stress capacity of this insect^31^. Some researchers also demonstrated that CDase can maintain the photoreceptor homeostasis of *Drosophila* in a cell non-autonomous manner^32^. Taken together, these studies suggested that CDases play important roles in cellular signaling and biological processes in insects.

The brown planthopper (BPH), *Nilaparvata lugens* (Stål), is the most economically important rice pest throughout Asian countries. Its outbreaks constitute a serious threat to the sustainability of rice yield^33^. The control of BPH depended heavily on the application of chemical insecticides and resistant rice varieties, which in turn influenced the growth and the population dynamics of BPH^34^. Moreover, food availability and environmental conditions are the critical challenges that impact the development of BPH during its long-distance migration. Previous studies suggested that nCDases could modify the biological responses elicited by external stimulus including the nutrient-deprivation and abiotic stresses^35^. In this study, we cloned a CDase gene, *NlnCDase*, from the brown planthopper. We expressed its protein in High Five cells and carried out enzymatic assays to determine its biochemical properties *in vitro*. Moreover, mRNA level and enzyme activity of NlnCDase at different life-stages, sex, and tissues were measured. We monitored the changes of mRNA level and enzyme activity of NlnCDase in BPH after the stimulation of stresses they frequently encounter during their lifespan, such as starvation, insecticide, resistant rice varieties and high temperature. Furthermore, the function of NlnCDase in the responses to starvation and temperature stresses was investigated through knocking down its transcripts by RNA interference (RNAi) technology.

## Result

### Sequence analysis of the *NlnCDase* gene

**Figure 1:**
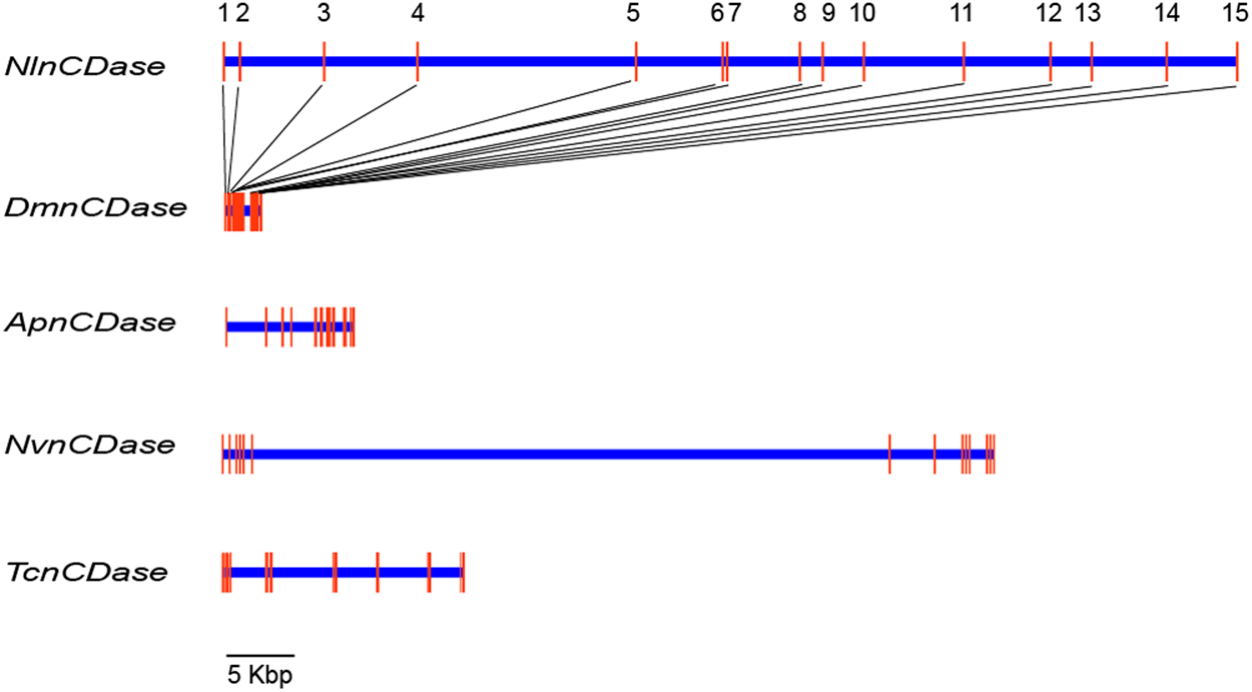
Genome structures of nCDases. Comparison of nCDase genes from five species of insects, *Nilaparvata lugens*, *Drosophila melanogaster*, *Acyrthosiphon pisum*, *Nasonia vitripennis* and *Tribolium castaneum*. Horizontal line represents the scaffold region, vertical boxes stand for the exons. The boxes between the line show the evolvement of exon segments between *NlnCDase* and *DmnCDase*.

**Figure 2:**
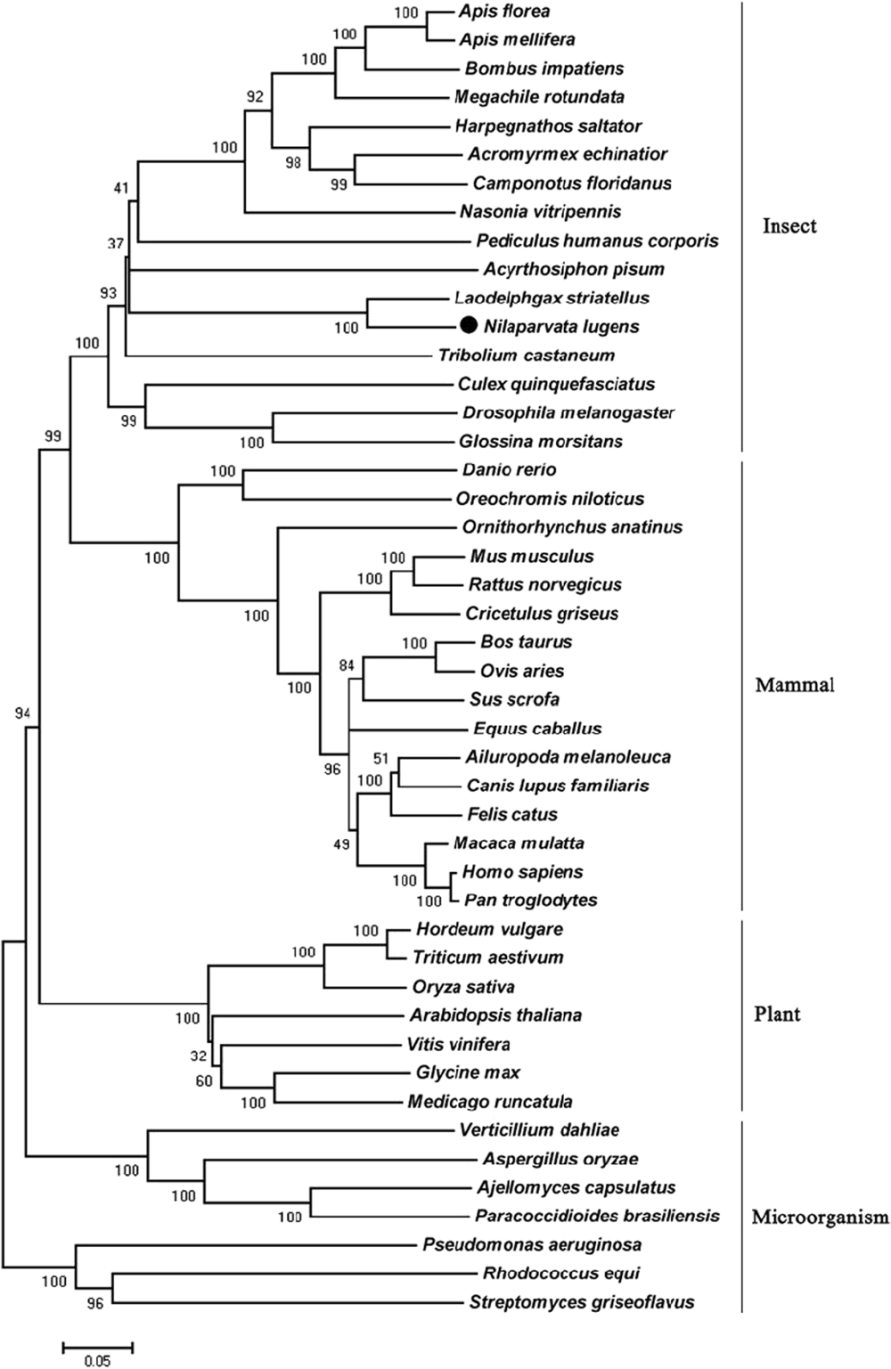
The phylogenetic relationship of neutral ceramidase homologues. The phylogeny reconstruction was generated by MEGA 5.0 with the neighbor-joining method with 1000 bootstrapping based on animo acid sequences. The numbers for the interior branches are bootstrap percentages. The following insects neutral ceramidase sequences were used: *Apis florea* (XP_003691475), *Apis mellifera* (XP_393129), *Bombus impatiens* (XP_003489963), *Megachile rotundata* (XP_003703614), *Harpegnathos saltator* (EFN86684), *Acromyrmex echinatior* (EGI57362), *Camponotus floridanus* (EFN68220), *Nasonia vitripennis* (XP_001606211), *Pediculus humanus* corporis (XP_002429644), *Acyrthosiphon pisum* (XP_001948595), *Laodelphgax striatellus* (JX569799), *Tribolium castaneum* (XP_968874), *Culex quinquefasciatus* (EDS45531), *Drosophila melanogaster* (BAC77635), *Glossina morsitans* (AFJ68095), *Danio rerio* (BAD69590), *Oreochromis niloticus* (XP_003449505), *Ornithorhynchus anatinus* (XP_001506611), *Mus musculus* (NP_061300), *Rattus norvegicus* (NP_446098), *Cricetulus griseus* (XP_003507579), *Bos taurus* (XP_002698412), *Ovis aries* (XP_004020325), *Sus scrofa* (XP_001924466), *Equus caballus* (XP_001501734), *Ailuropoda melanoleuca* (XP_002914440), *Canis lupus* familiaris (XP_543587), *Felis catus* (XP_003993931), *Macaca mulatta* (XP_001100516), *Homo sapiens* (NP_063946), *Pan troglodytes* (XP_507791), *Hordeum vulgare* (ACI00279), *Triticum aestivum* (ABX76295), *Oryza sativa* (ACA49516), *Arabidopsis thaliana* (AEC09477), *Vitis vinifera* (XP_002277379), *Glycine max* (XP_003530830), *Medicago truncatula* (XP_003617915), *Verticillium dahlia* (EGY17874), *Aspergillus oryzae* (XP_001818969), *Ajellomyces capsulatus* (EEH08915), *Paracoccidioides brasiliensis* (EEH43578), *Pseudomonas Aeruginosa* (2ZWS), *Rhodococcus equi* (ZP_08154631), *Streptomyces griseoflavus* (EFL38370).

The full-length *NlnCDase* cDNA sequence is 2663 bp, including a 72-bp 5′-UTR, a 2193-bp ORF, and a 398-bp 3′-UTR. It encodes a deduced protein of 730 amino acids, with a putative signal peptide of 25 residues at the N-terminal (Supplementary Fig. S1). The Mw and pI of the deduced protein is 80,212.6 Da and 5.85, respectively. In the genome of *N*. *lugens*^36^, the *NlnCDase* gene spanned 74,291 bp of the genome, with 15 exons ranging from 85 to 491 bp, and these exons were interrupted by 14 introns (Fig. 1).Generally the 5′ boundary of introns contained a dinucleotide GT (donor site), while the 3′ boundary of introns contained a dinucleotide AG (acceptor site)^37^. While several other types of splice dinucleotides were also found in the human genome except GT-AG dinucleotides (splice dinucleotides)^38^. Except for the 6^th^ and 14^th^ introns, all introns started with GT and ended with AG^39^, suggesting a good conformance with the ‘gt/ag’ rule (Supplementary Table S1). The exons of the *NlnCDase* gene had higher variety in their position and size than those from other insect species. Amino acid sequence alignment showed the NlnCDase protein has a 47.34%, 49.38%, 39.82%, 37.82% sequence identity to the nCDases from *D*. *melanogaster*, *T*. *castaneum*, *D*. *rerio*, *H*. *sapiens* respectively. The highly conserved amidase domain (NXGDVSPNXXG^P^/_X_XC) was found in the NlnCDase protein (Supplementary Fig. S2), suggesting its role in the hydrolytic reaction^40^. Phylogenetic analysis results showed that the nCDases were mainly clustered into four subclasses, insects, mammals, plants, and microorganisms based on their origins (Fig. 2).

### Enzymatic and biochemical characteristics

**Figure 3:**
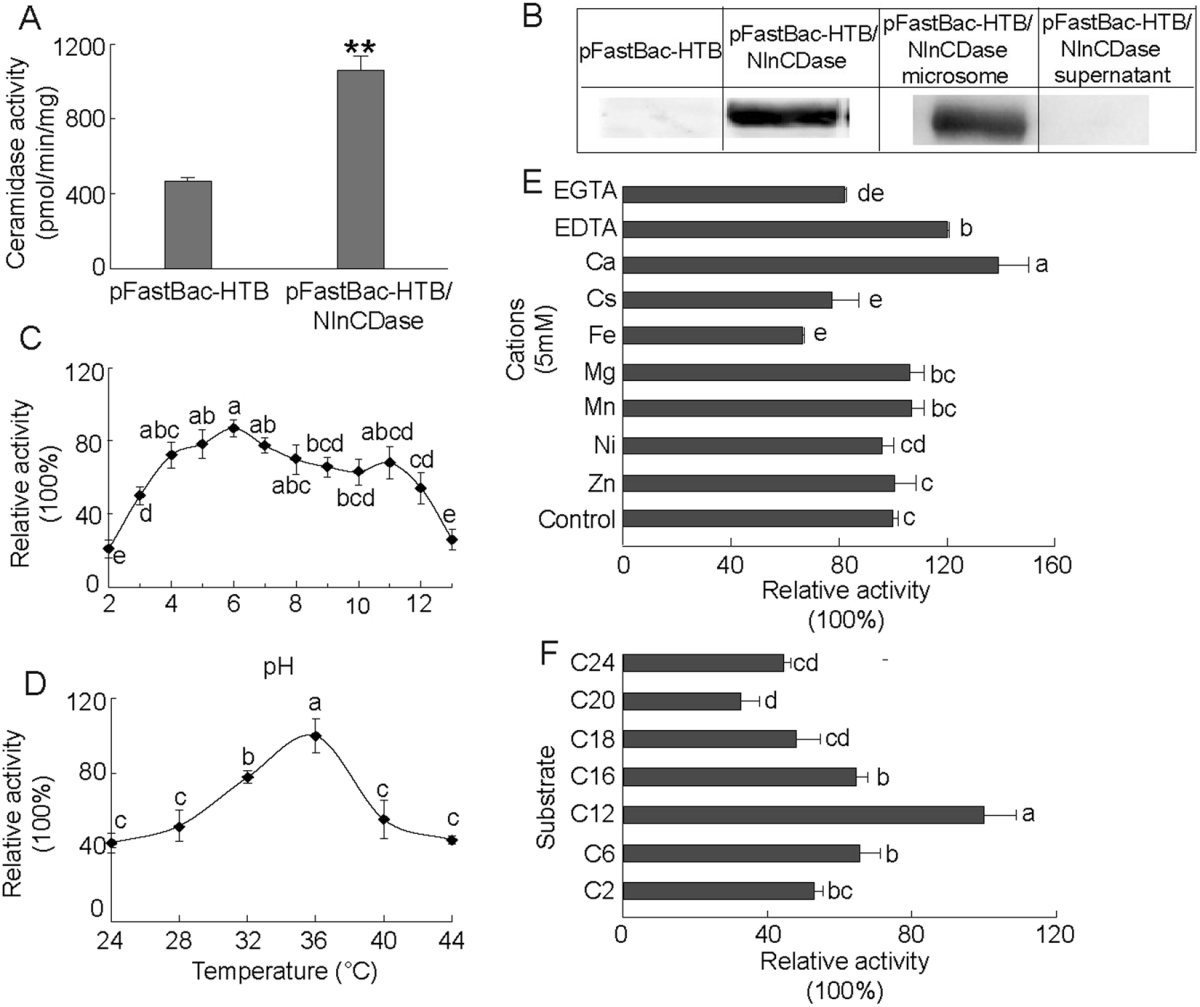
Biochemical properties of *Nilaparvata lugens* nCDase (NlnCDase). (**A**) Microsomes isolated from High Five cells transfected with pFast-HTB or pFast-HTB/NlnCDase were assayed for ceramidase activity. (**B**) The microsomes and supernatant of pFast-HTB/NlnCDase were subjected on SDS-PAGE gel and analyzed by western blotting. The relative Mr. was estimated according to protein standards. (**C**) The NlnCDase activity was assayed at different pH values. The pH was adjusted by adding the following buffer: Acetate (pH 2–6), Tris (pH 7–8), Glycine (pH 9–13). Ceramidase activity of the NlnCDase at each pH was calculated by subtracting ceramidase activity in pFast-HTB microsomes from that in pFast-HTB/NlnCDase microsomes. The NlnCDase activity at pH 6.0 was highest and sets as 100%, and ceramidase activity at other pH values was expressed as % of the maximal activity. (**D**) Optimum temperature of NlnCDase. The NlnCDase activity at each temperature was conducted at pH 6.0. The NlnCDase activity at 36 °C was highest and set as 100%, and ceramidase activity at other temperature was expressed as % of the maximal activity. (**E**) Effects of different cations on NlnCDase activity. 5 mM indicated cations were added in reaction buffer. The NlnCDase activity with Ca^2+^ added was highest and set as 100%, and ceramidase activity with different cations values was expressed as % of the maximal activity. (**F**) Substrate specificity of NlnCDase. The NlnCDase activity was highest with substrate of C_12_ ceramide and set as 100%, and ceramidase activity on other substrates was expressed as % of the maximum activity. All data represent the mean value ± SE of three independent experiments performed in duplicate, and the data with different letters are significantly different (two-samples *t* test, *P* < 0.05).

The *in vitro* activity assay was carried out to investigate the function of NlnCDase. The pFastBac-HTB/NlnCDase produced ~2-fold higher level of SPH than the control (pFastBac-HTB) (Fig. 3A), suggesting NlnCDase’s role in hydrolyzing ceramides into sphingosines. According to the western blot analysis result, the NlnCDase protein in High Five cells was detected in the cellular microsomes, instead of in the cellular supernatant (Fig. 3B). To further investigate the physiological property of NlnCDase, we defined the subcellular localization of this enzyme. Totally we checked four cellular organelles including plasma membrane (PM), mitochondria (MR), lysosome (Lyso) and endoplasmic reticulum (ER). Although we did not observe the fusion yellow fluorescence signal in any of those organelles, the result clearly suggested that NlnCDase may be localized in other organelles instead of the four we detected (Supplementary Fig. S4). The NlnCDase enzyme had a broad pH range for its activity, and its optimal pH was at 6.0 (Fig. 3C), suggesting that it might be an nCDase *in vitro*. The NlnCDase displayed an optimal temperature of around 36 °C (Fig. 3D). Moreover, the relative enzymatic activity of NlnCDase was inhibited by EGTA, Cs^+^, and Fe^2+^, while activated by EDTA or Ca^2+^. And it was not affected by Mg^2+^, Mn^2+^, Ni^+^, and Zn^2+^ (Fig. 3E). In addition, according to the substrate specificity assay, NlnCDase showed a broad capacity to hydrolyze different ceramide varieties and had a preference for the short and medium chain ceramide, with the substrate preference: C_12_- ceramide >C_6_- ceramide >C_16_- ceramide. It showed low activity for the very-short chain C_2_- ceramide and the long chain C_20_- ceramide, C_24_- ceramide (Fig. 3F).

### The MTT assay of NlnCDase *in vitro*

To determine the effect of NlnCDase on the cell proliferation, the MTT assay was conducted. The dye MTT [3-(4,5-dimethyl-2-thiazolyl)-2,5-diphenyl-2-H-tetrazolium bromide] was reduced by succinate dehydrogenase (SDH), which existed only in the mitochondria of live cells. So the MTT assay was always used to check the viability of cells and the OD value was a widely adopted indicator for the comparison of cell growth^41^. After the cell transfection, the OD value increased more slowly in NlnCDase - overexpressed (pFastBac - HTB/NlnCDase) group than the control (pFastBac - HTB) group, indicating a lower cell growth rate in the former group than the later (Supplementary Fig. S5).

### Temporal and spatial expression patterns of *NlnCDase* gene

**Figure 4:**
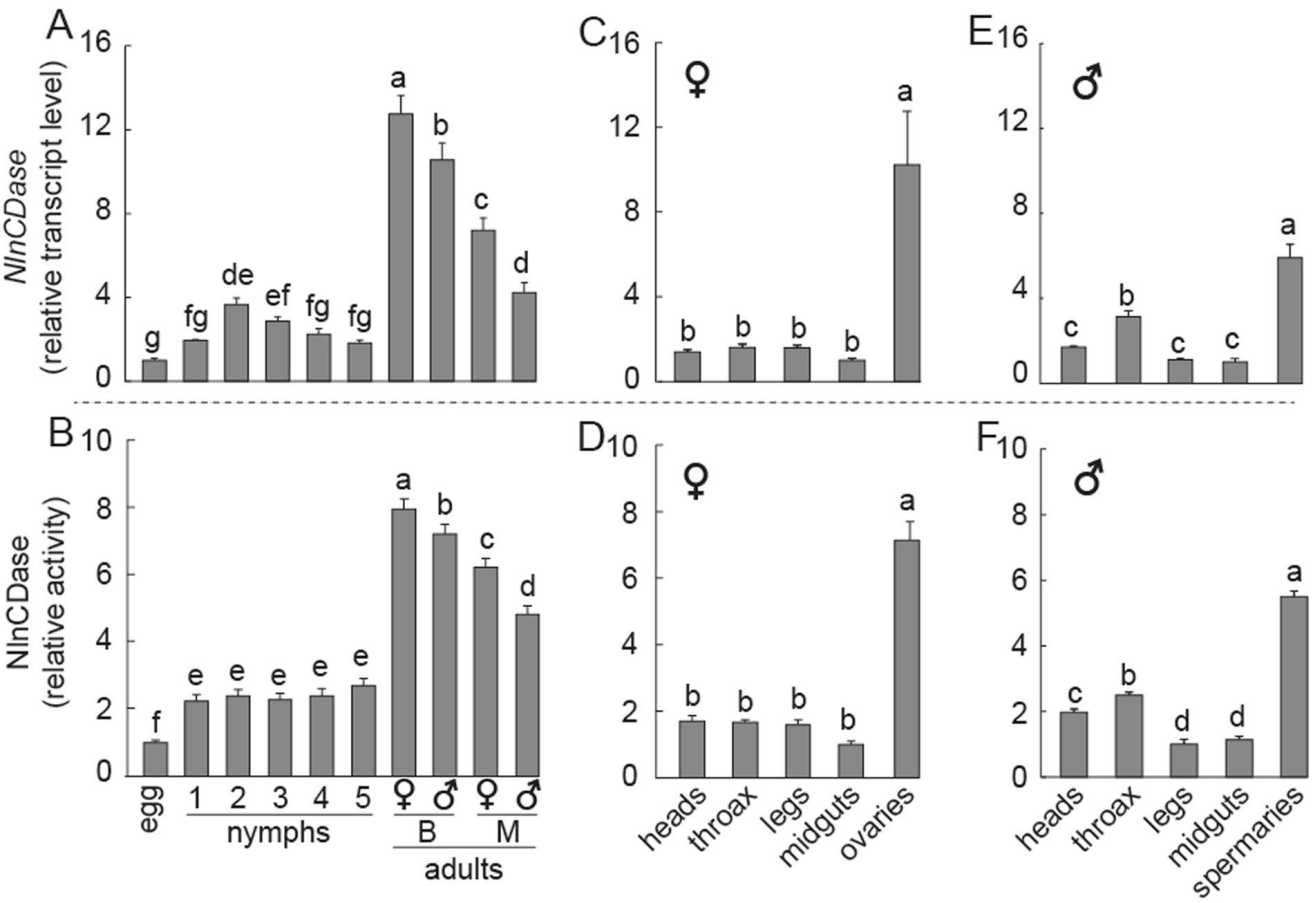
Relative transcript level and relative activity of NlnCDase in different stages, sexes, wing types and tissues. (**A**) Relative transcript level of *NlnCDase* in different life stages, different sexes and different wing types. (**B**) Relative activity of NlnCDase in different life stages, different sexes and different wing types. (**C**) Relative transcript level of female *NlnCDase* in different tissues. (**D**) Relative activity of female NlnCDase in different tissues (**E**) Relative transcript level of male *NlnCDase* in different tissues. (**F**) Relative activity of male NlnCDase in different tissues. The relative expression level was expressed as mean ± SE (N = 3). Different alphabets (a, b, c etc.) above the columns indicate the significant differences at the P < 0.05 level. Similar lowercase letters above the columns indicate no significant difference detected. 1: first instar nymph; 2: second instar nymph; 3: third instar nymph; 4: fourth instar nymph; 5: fifth instar nymph; ♀: female; ♂: male; M: macropterous adults; B: brachypterous adults.

To better understand the expression profile of *NlnCDase* gene *in vivo*, we monitored its expression in different growth stages and tissues of BPH using qRT-PCR. The results showed that the relative gene transcript accumulation was significantly increased in BPH adults than in eggs and nymphs. No significant difference was observed in the transcript levels of nymphal stages studied. Females showed higher transcript level of *NlnCDase* gene than males. And transcript levels in the brachypterous adults were markedly higher than those in the macropterous adults. The female brachypterous adults had the highest transcript level of *NlnCDase* among all BPH stages tested (Fig. 4A). Furthermore, BPH female and male adults were dissected to measure the tissue-specific transcript accumulation levels of *NlnCDase*. The highest transcript levels were found in two reproductive organs, the ovaries and the spermaries. We did not observe significant transcript differences among the other body parts (Fig. 4C,E).

### The NlnCDase relative activity *in vivo*

The time- and spatial-specific expression of *NlnCDase* gene in BPH indicated that the activity of NlnCDase enzyme might also be different among different tissues and developmental stages. To confirm this, we further analyzed the relative activity of NlnCDase enzyme *in vivo*. The relative activity of NlnCDase enzyme also exhibits higher levels in the adults and reproductive organs (Fig. 4B,D,F), which correlated well with the transcript level of *NlnCDase* gene.

### *NlnCDase* gene expression and NlnCDase enzyme relative activity in response to stresses

**Figure 5:**
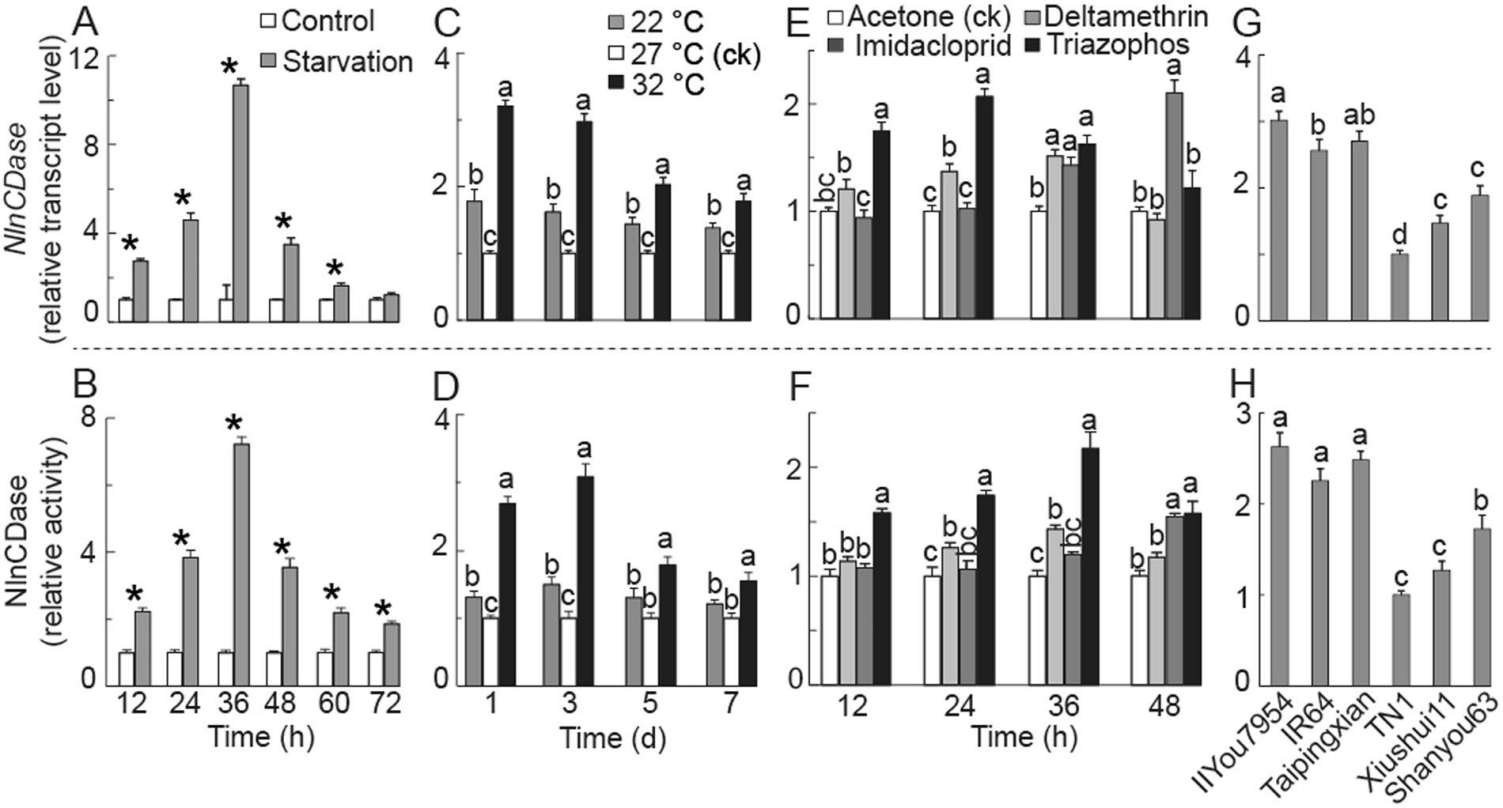
Relative transcript level and relative activity of NlnCDase after exposure to stresses. (**A**) Relative transcript level of *NlnCDase* after treated with starvation. (**B**) Relative activity of NlnCDase after treated with starvation. (**C**) Relative transcript level of *NlnCDase* after treated with abnormal temperature. (**D**) Relative activity of NlnCDase after treated with abnormal temperature. (**E**) Relative transcript level of *NlnCDase* after treated with insecticides. (**F**) Relative activity of NlnCDase after treated with insecticides. The acetone was used as ck. (**G**) Relative transcript level of *NlnCDase* after fed with different rice varieties. (**H**) Relative activity of NlnCDase after fed with different rice varieties. The data was expressed as mean ± SE (N = 3). Significance between the columns is shown by different alphabets (a, b, c etc.) or ‘*’ at the *P* < 0.05 level. Similar lowercase letters above the columns indicate no significant difference detected.

To investigate how the NlnCDase responded to the abiotic and biotic stresses with which BPH frequently encountered, we further used the transcript level and the relative enzyme activity as the two indicators to monitor the change of NlnCDase in stressed BPH. Both indicators were significantly increased after the starvation treatment. Within 12 h after the starvation, the two indicators raised ~3 times in starved insects in comparing to the untreated group. Both indicators were elevated in the first 36 h with more than 10 folds in transcript levels and 7 folds in the enzymatic activity of NlnCDase. And they reached the highest level at 36 h, which later decreased to the normal level in the next 36 h (Fig. 5A,B).

The second abiotic stress we used for BPH treatment is the temperature. Both indicators were up-regulated when BPH were treated with high or low temperature (Fig. 5C,D). The increase of these indicators was more significant within the first 3 days under temperature stress. And the changes of two indicators were greater in BPH exposed to high temperature than those exposed to low temperature. Under the stress of low temperature, the transcript level of *NlnCDase* gene was significantly increased in all days examined, whilst the enzyme activity only showed significant increase on the 1^st^ and 3^rd^ days of stress induction. In high temperature stressed BPH, however, both two indicators were significantly different from control in all test days.

The next abiotic stresses we used for BPH treatment are the insecticides normally used for control of BPH in Asia. The two indicators also increased in BPH after exposing to the three insecticides: deltamethrin, imidacloprid, and triazophos (Fig. 5E,F). 24 h and 36 h after the deltamethrin exposing, the two indicators in BPH are significantly increased. Unlike deltamethrin, the effect of imidacloprid on the level of the indicators slowly occurred (36 h after the treatment). Triazophos had faster and longer effects on the two indicators than the other two insecticides. Its effects were significant at 12 h after treatment, reached the strongest at 24 h, and lasted until 48 h.

**Figure 6:**
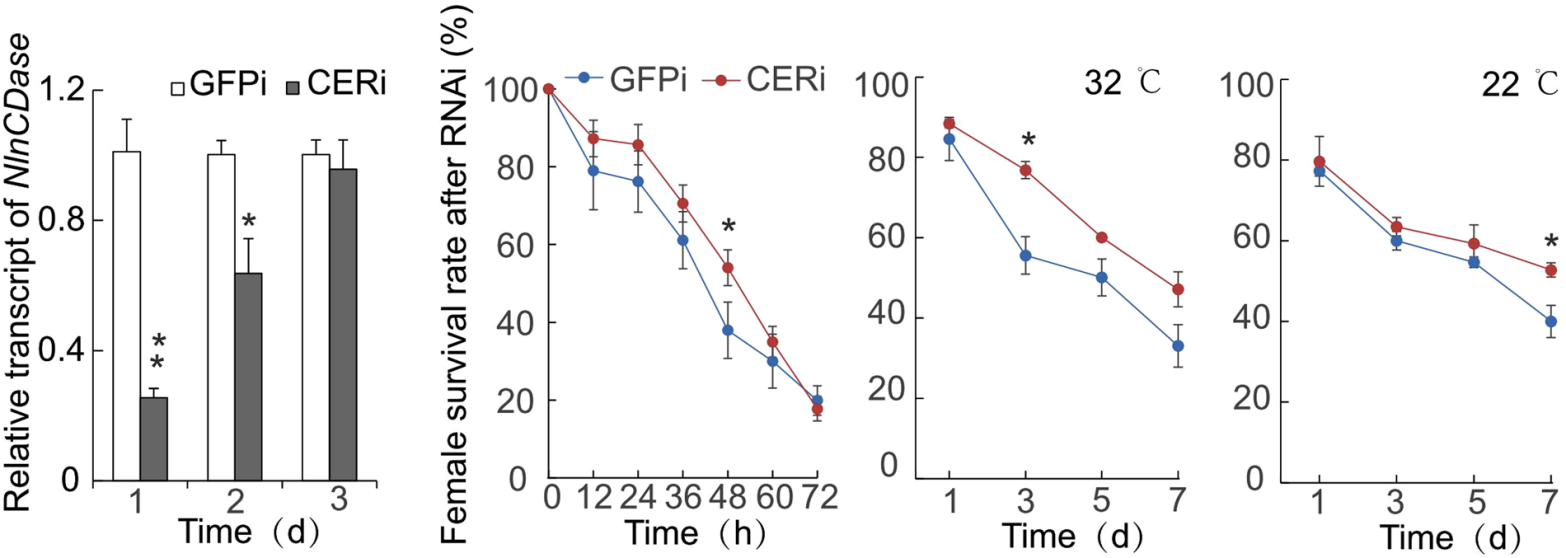
Bioassay of BPH female adults under the exposure of stresses after RNAi. (**A**) Relative transcript level of *NlnCDase* after ds*GFP* (GFPi) and ds*NlnCDase* (CERi) injection. (**B**) The female survival rate after starvation treatment. (**C**) The female survival rate after reared in high temperature (32°C). (**D**) The female survival rate after reared in low temperature (22°C). The data was expressed as mean ± SE (N = 3). Significance is shown as follows: **P < 0.01; *P < 0.05.

The biotic stress we used for BPH treatment was the rice varieties which showed different resistance to BPH. The resistance of rice was previously measured (unpublished data) following the method of Heinrichs’s^42^. The two indicators were significantly elevated in BPH after feeding on the resistant varieties (II You 7954, IR 64, and Tai Ping Xian) (Fig. 6G,H). However, the NlnCDase activity was not different among BPH stressed with different resistant varieties. In susceptible rice-feeding treatment, the BPH fed with TN1 had the lowest levels of the two indicators.

### Knocking down *NlnCDase* increased the survival rate of BPH

To further investigate the function of *NlnCDase* in BPH, we knocked down *NlnCDase* by RNAi technology. The transcript of *NlnCDase* was significantly decreased on the first day after *NlnCDase-*dsRNA injection, and the decrease maintained for at least two days (Fig. 6A). Under the starvation stress, the female adults with lower *NlnCDase* transcripts had a better survival than the control group. At 48 h after the starvation, the survival rate was significantly different between the *NlnCDase-*dsRNA injected and *GFP*-dsRNA injected groups (Fig. 6B). Under abnormal temperature, the injection of *NlnCDase*-dsRNA significantly increased the survival rate of BPH on the third day of high temperature treatment, while the increase happened on the seventh day under low temperature treatment (Fig. 6C,D). In conclusion, the female adults survived better under abiotic stress after the knocking down of *NlnCDase*.

## Discussion

In the current study, we cloned and characterized an nCDase from an important rice pest *Nilaparvata lugens*. The conserved protein sequences between *NlnCDase* and its homologues from other animals indicated their fidelity in the metabolizing of ceramides. It was not surprised to see that the amidase domain of NlnCDase was also similar to those of nCDases from other organisms including animals, plants and fungus, since it has been proved to be essential for the activity of nCDase in human^43,44^. Phylogenetic analysis showed that *NlnCDase* gene was more conserved with the *nCDases* from eukaryotes.

The CDase of *N*. *lugens* exhibited a broad pH property and was named as *NlnCDase* based on its optimal PH (pH~6.0), while the nCDases from the two insects *T*. *castaneum* and *L*. *striatellus* showed acidic (pH ~ 5.0) and alkaline (pH~8.0) pH optimum respectively^29,30^, suggested that closely related nCDases had different pH optimum in different organisms. For instance, then CDases from fruit fly^17^, mouse^16^, rat^20^, zebrafish^21^, and human^22^ have a neutral pH optimum, while their homologue from *D*. *discoideum* showed an acidic pH optimum of 3.5^27,29^. The optimized PH of CDases was suggested to depend on their micro-environments. In humans, CDases were found to be localized in different compartments, including mitochondria (MR), lysosome (Lyso), endoplasmic reticulum (ER) and Golgi complex^45,46^. In rats, the insoluble nCDase in kidney was localized at apical membranes of kidney tubules^20^. The nCDase of *Arabdoposis* localized predominantly on the ER^25^. In insects, both nCDases from *T*. *castaneum* and *L*. *striatellus* were localized in the plasma membrane (PM)^29^. Interestingly, we detected the expression of NlnCDase in the microsomes of High Five cells, and did not localize the protein in either four organelles we examined, suggesting that NlnCDase might be expressed in other microsomes, rather than the ones we tested. And we proposed the hypothesis that the broad expression of NlnCDase may fit its broad biological functions as well.

NlnCDase has similar bio-characteristics to nCDases from *T*. *castaneum* and *L*. *striatellus*^29,30^, both in estimated molecular mass and in optimized temperature. The optimum temperature for nCDase enzyme *in vitro* for all three insects is around 36 °C, different from the rearing temperature for those insects. Moreover, the enzymatic activity could be affected by various ions which might be absorbed by the insects from the environment. The NlnCDase activity was enhanced by EDTA and Ca^2+^ while inhibited by EGTA, Cs^+^ and Fe^2+^. The result was consisted with previous researches showing that Fe^2+^ or Fe^3+^ decrease the activity of nCDase from *T*. *castaneum*, *L*. *striatellus*, rice, human, and fruitfly^17,29,30,40,47^. Our research found that NlnCDase can metabolize ceramides with very-short to very-long carbon acyl-chain, whilst it preferred the short and medium chain ceramides. This subtract preferences of NlnCDase was consisted with those of the nCDase from *L*. *striatellus*^30^, while different with those of the nCDase from *T*. *castaneum*^29^. Ceramides with different chain lengths of the fatty acyl have different biophysical functions^48,49^. The capacity of hydrolyzation to different ceramides indicated the essential roles of NlnCDase in BPH, which remains to be characterized in further studies.

BPH is a metamorphosis herbivore. Its macropterous adults have the capacity of long-distance migration^50,51^, while the brachypterous adults have higher fecundity. Similar to the findings in *T*. *castaneum* and *L*. *striatellus*, *NlnCDase* is ubiquitously expressed and significantly enriched in the adults of BPH, especially in the two reproductive organs, ovaries and spermaries^29,30^. While in *D*. *melanogaster*, alkaline CDase’s mRNA level was highest in pupal stage, which consisted with the observation that the inactivation of this enzyme prolonged the pre-adult time^31^. In BPH, the ovaries had higher NlnCDase than spermaries, and brachypterous female adults had higher NlnCDase than macropterous adults, suggesting that more NlnCDase was required for the fertilization of brachypterous BPH. Further investigations are needed to test this hypothesis.

Ceramides act as essential components of eukaryotic membranes and the second messengers in the organism^12^, which levels were required to stay homeostasis. Recent research demonstrated that up-regulated aCDase was required for the acute myeloid leukemia (AML) blast survival^52^. The sphingolipid dysregulation through decreased ceramide levels and elevated sphingosine 1-phosphate, which mediated by aCDase, promoted cancer cell growth and survival. In our study, over-expressed NlnCDase arrested the proliferation of cells in MTT assay. The overexpression of NlnCDase may affect the degrading of ceramides and the accumulation of sphingosines and S1P. Not only the decrease of ceramides may affect the proliferation of cells, but also SPH and S1P were previously found to arrest cell growth^53^.

Moreover, the up-regulation of CDase mRNA and protein can be induced by some cytokines [e.g. interferon c, interleukin 1b (IL-1b), tumour necrosis factor a (TNFa)], cytotoxic agents (e.g. chemotherapeutic agents and modulators of multidrug resistance) and environmental stresses (e.g. heat shock, ionizing and ultraviolet radiation, and growth-factor withdrawal)^9^. Previously, the levels of ceramides were found to be increased after environmental stress. For instance, the level of ceramides was increased after heat shock treatment in mouse and yeast^54,55^. Exposing brain cells to oxidative stress also increased the accumulation of ceramides^56^. INS-1 cells enhanced the CDase activity through secreting exosomes containing nCDase to reduce a high concentration of ceramides-induced cell death^57^. In plants, the transcripts level of wheat nCDase was strongly induced by infection of stripe rust fungus^24^. nCDase activity was also higher in rice stripe virus (RSV)-infected *L*. *striatells*^30^. According to our study, both the transcript level and enzymatic activity of NlnCDase were raised in BPH under various environmental stresses (e.g. starvation, high temperature, pesticides treatment, and resistant rice varieties). NlnCDase showed higher induction in response to high temperature than to low temperature in our study, however, it should not be ruled out that this enzyme may also have stronger induction to more severe cold shock that we did not use in the current study. It might be interesting to see if the accumulation of ceramides induced by environmental stresses stimulated the induction of NlnCDase, or *vice versa*.

When *NlnCDase* was knocked down by RNAi, the BPH exhibited higher survival to the starvation and temperature stresses, indicating that increased expression of *NlnCDase* gene may reduce the ability of BPH to adapt to certain forms of stress. Since an increase in the expression of *NlnCDase* gene may reduce the levels of its substracts, the ceramides, we postulated that a decrease in the levels of ceramides may render a susceptibility to stress. Ceramides have been implicated in cell growth arrest and death in mammalian or in response to various forms of stress in insect cells. However, increasing studies demonstrate that basal levels of ceramides are also important for fitness of animals because knocking out some ceramide synthase genes (*CerS*) caused various pathological effects in mice. For example, knocking out *Cers1* causes cerebellar neurodegeneration, due to the death of Purkinje cells^58^. These results suggest that the protective role of ceramides in stress responses is conserved between insects and mammals.

In conclusion, NlnCDase may mediate the ceramides balance in BPH and this enzyme may play important roles in the development and stress responses of BPH through regulating the biosynthesis of sphingolipids. Considering the limited information concerning CDase’s role in invertebrate species, especially in insects, our study may improve the understanding of CDase’s functions in insects.

## Materials and Methods

### Brown plant hoppers and rice seedlings

The laboratory strain of BPH that was used in all experiments originated from a field population in the Huajiachi campus of Zhejiang University, Hangzhou, China. The insects were reared on susceptible rice seedlings cv. Taichung Native 1 (TN1) at 27±1 °C, 70% relative humidity and a 16:8 h light:dark photoperiod. Three tolerant rice varieties to BPH (IR 64, TaiPing xian and II You 7954) and three susceptible rice varieties to BPH (TN1, Xiushui 11, Shanyou 63) were kept in our laboratory.

### cDNA cloning

A BLAST search of the BPH transcriptome data (SRA accession number SRX023419) using fruit fly nCDase gene (BAC77635) as query revealed a partial cDNA sequence of *NlnCDase*. Total RNA was extracted from 6 adult BPH using TRizol reagent (Invitrogen) according to manufacturer’s instructions. RNA integrity was checked by separating on a 1% agarose gel and staining with ethidium bromide. The quantity of RNA was determined by measuring OD_260_ nm with the NanoDrop2000 Spectrophotometer (ThermoScientific).First-strand cDNA was synthesized by PrimeScript^TM^ 1^st^ Strand cDNA Synthesis Kit (Takara). The open reading frame of the *NlnCDase* gene was confirmed by performing RT-PCR and the RACE PCR. In order to clone the full length cDNA sequence, 5′-RACE and 3′-RACE were carried out separately using the 5′-Full RACE Kit and 3′-RACE Core Set Ver. 2.0 (TaKaRa) with the following gene-specific primers (5′ RACE: 5′-ATTCTGAAGGTAGGACTGCGGACTG-3′; 3′ RACE: 5′-TGGGCACAAGTGAAGCCAAAGT-3′). The primers were designed according to the partial fragment mentioned above. The full-length *NlnCDase* gene was generated by PCR with a sense primer with a HindIII site (underlined) (5′-CCGCTCGAGATGACTCAACACACA-3′) and an anti-sense primer with a XhoI site (double underlined) (5′-CCCAAGCTTTCACAATGTTATTCCTC-3′) under the conditions of denaturing at 94 °C for 3 min, 35 cycles of denaturing at 94 °C for 30 s, annealing at 62 °C for 30 s, extension at 72 °C for 2.5 min and a final extension step at 72 °C for 10 min. The amplified product was separated onto 1% agarose gel and purified using the AxyPrep™ DNA Gel Extraction Kit (Axygen). The purified PCR product containing the putative *NlnCDase* sequence was routinely cloned into the pGEM-T Easy vector (Promega), and then sequenced to confirm the correctness.

### Bioinformatics analysis

The mRNA (cDNA)-genome alignments were performed with the program Spidey (http://www.ncbi.nlm.nih.gov/spidey/). The theoretical molecular weight (Mw) and isoelectric point (Ip) of the NlnCDase were predicted by using ExPASy server (http://www.expasy.org/tools/protparam.html). Signal peptide was predicted by SignalP 4.1 Server (http://www.cbs.dtu.dk/services/SignalP). The predicted amino acid sequence of the *NlnCDase* gene was used as a query sequence to search sequences in National Center for Biotechnology Information (NCBI) with BLAST net server (http://blast.ncbi.nlm.nih.gov/Blast.cgi). The amino acid multiple sequence alignment of nCDase was performed by using Clustal W2 (http://www.ebi.ac.uk/.Tools/msa/clustalw2/). Phylogenetic analysis based on the amino acid sequences was conducted to investigate the relationship between NlnCDase and nCDases of other species. A phylogenetic tree was constructed by MEGA 5 software^59^ with the neighbor-joining method and bootstrapping sampled 1,000 replicates.

### NlnCDase overexpression in High Five Cells

The *NlnCDase* gene’s cDNA was sub-cloned into the corresponding sites of transfer vector pFast-HTB. The resulting NlnCDase expression construct (pFast-HTB/NlnCDase) was transformed into competent DH10Bac *Escherichia coli* (Invitrogen) for transposition into the bacmid according to the manufacturer’s instruction to obtain the recombinant bacmid used for transfection. PCR analysis was carried to verify the presence of the *NlnCDase* gene in the recombinant bacmid. High Five cell line (Tn5B-1-4),originated from the ovarian cells of the cabbage looper, *Trichoplusia ni*, was cultured in our laboratory at 28 °C in TNM-FH Insect Medium (Sigma-Aldrich) supplemented with 10% (v/v) heat-inactivated fetal bovine serum (Invitrogen). The confirmed recombinant bacmids of pFast-HTB/NlnCDase and pFast-HTB were transfected with lipofectamine 2000 (Invitrogen) into High Five cells to obtain recombinant baculovirus carrying the *NlnCDase* ORF and control baculovirus respectively. For transient expression of recombinant NlnCDase protein for assay, High Five cells were infected with infectious medium containing phage for two days.

### Microsomes preparation

The High Five cells were collected by centrifugation at 2,000 *g* for 10 min, and washed two times with ice-cold PBS. Cells were sonicated on ice in lysis buffer (20 mM Tris-HCl, 0.25 M Sucrose, pH 7.4) containing 20 mg/ml protease inhibitor (Roche Applied Science). The total cell lysates were centrifuged at 1,000 *g* for 10 min at 4 °C to remove nuclear fraction and unbroken cells, and the post-nuclear supernatant was centrifuge at 100,000 *g* for 1 h at 4 °C to obtain microsomes. The supernatant was collected and the fraction was dissolved in assay buffer (25 mM Tris-HCl, pH 7.4).

### SDS-PAGE and Western blotting analysis

The concentration of total proteins was determined using Pierce BCA Protein Assay Kits (Thermo scientific). 40 μg of proteins were separated on 10% Sodium dodecyl sulfate-polyacrylamide gel electrophoresis (SDS-PAGE) as previously reported^60^ and transferred to a nitrocellulose membrane by semi-dry electrophoresis. After blocking with 5% skimmed milk overnight at 4 °C, the membrane was probed with anti-His antibody (diluted 1:1000), followed by incubating with anti-mouse IgG-HRP antibody (diluted 1:2000) as a secondary antibody. The HRP-labeled antibody was detected using SuperSignal West Pico Chemiluminescent Substrate kit (Thermo scientific) according to manufacturer’s instruction. Chemifluorescent signal was detected by FluorChem^®^FC2 (Alpha Innotech). The pre-stained protein marker (Thermo Fisher Scientific) was used as a molecular weight standard.

### CDase biochemical characterization

CDase activity was determined by the release of sphingosines from ceramides. The NlnCDase protein was prepared by the Bac-to-Bac expression system, whose expression (a band of ~90 kDa) was further confirmed by the western blotting. The enzyme activity assay was conducted by detecting the C18-sphingosine content after the hydrolysis reaction of the C12 ceramide, which was used as the substrate for NlnCDase. D-e-C17 sphingosine and D-e-C18 sphingosine were used as the internal and the external standards for the products, respectively. We used the retention time to assist the chemical detection of products, and it was at 11.7 min and 15.3 min for D-e-C17 and D-e-C18 sphingosine, respectively. To verify whether NlnCDase protein has CDase activity, we separately prepared microsomes from High Five cells transfectted with pFastBac-HTB/NlnCDase and pFastBac-HTB to carry the degradation reactions. The CDase activity assay was performed at pH 7.5, 37 °C using C12-ceramide (d18:1/12:0, *N*-lauroyl-D-*erythro*-sphingosine) as a substrate essentially following our previous study^31^. Then the biochemical characteristics of this enzyme were tested *in vitro*. The optimal conditions of this enzyme were measured, including different pH values ranging from 2.0 to 13.0, different temperature ranging from 24 °C to 44 °C, dependence of various cations (5 mM) including Mn^2+^, Fe^2+^, Zn^2+^, Ca^2+^, Mg^2+^, Ni^2+^, Cs^+^, EDTA and EGTA, and preference of substrates, i.e., C_2_- ceramide (N-acetoyl-D-*erythro*-sphingosine), C_6_- ceramide (N-Hexanoyl-D-*erythro*- sphingosine), C_12_- ceramide (N-lauroyl- D-*erythro*-sphingosine), C_16_- ceramide (N-palmitoyl-D-*erythro*-sphingosine), C_18_- ceramide (N-acetoyl -D-*erythro*-sphingosine), C_20_- ceramide (N-arachidoyl-D-*erythro*-sphingosine) and C_24_- ceramide (N-lignoceroyl-D-*erythro*-sphingosine).

### Subcellular localization in the High Five cell

*NlnCDase* was tagged with enhanced green fluorescent protein eGFP in the N terminal and C terminal separately to construct fusion proteins of eGFP-*NlnCDase* and *NlnCDase*-eGFP in the High Five cells. The bacmid containing pFast-HTB-eGFP/*NlnCDase* and pFast-HTB-*NlnCDase*/eGFP were transfected into High Five cells to overexpress the fusion proteins, and recombinant baculoviruses were harvested to re-infect High Five cells for24 h. The infected cells were separately stained with PM dye[1,1′-dioctadecyl-3,3,3′,3′-tetramethylindocarbocyanine perchlorate (DiIC18(3))], mitochondria dye (Mito-Tracker), lysosome dye (Lyso-Tracker Red), and endoplasmic reticulum dye (ER-Tracker Red) after fixing with 4% paraformaldehyde. All these dyes were purchased from Biyuntian (http://www.beyotime.com/index.htm), China. All those organelles were in red fluorescence while the NlnCDase-eGFP and eGFP-NlnCDase fusion proteins in green under the observation of confocal microscope (Leica TCS SP5, Germany).

### MTT assay

96-well plates, each well inoculated with about 1500 cells, were prepared before re-infecting with recombinant baculoviruses of pFast-HTB- *NlnCDase* and pFast-HTB.10 ul MTT solution was added into 96-wells and cultivated for 4 h before the addition of 100 ul formanzan solution. Cells were continually cultivated until formanzan solution was absolutely dissolved before being examined at 570 nm by microplate reader. Because the cell numbers had liner correspondence with OD values in MTT assay, the OD values were recorded to estimate the proliferation of High Five Cells.

### Expression pattern and relative enzyme activity of NlnCDase

Different developmental stages and sexes sampled in this study included eggs, the 1^st^ to 5^th^ instar nymphs, macropterous female and male adults as well as brachypterous female and male adults. The fresh weight of each sample was about 0.1 g. The organs were dissected on ice from4^th^ day old macropterous female or male adults (about 50), including heads, thoraxes, legs, midguts, ovaries and spermaries. Total RNA was extracted according to the protocols mentioned above, and then reverse transcribed into cDNA using PrimeScript^®^ RT reagent Kit (Perfect Real Time) (Takara). The cDNA was reversely transcribed from total RNA isolated from BPH of different stages, sexes and tissues. The primer sequences used in qPCR were NqPCRF (5′-AGTCCTACCTTCAGAATC-3′) and NqPCRR (5′-TTCGTGTTGTTCATACTG-3′) for *NlnCDase* gene, and alpha-tubulin F(5′ ACGTCCTTGGGAACGACATC-3′) and alpha-tubulin R(5′ GCTTTGAGCCAGACAACCAAA-3′) for *alpha-tubulin* house-keeping gene as an internal control. The qPCR was run on an ABI 7300 Real-Time PCR System (Applied Biosystems, Branchburg, NJ) with the SYBR^®^Premix Ex Taq™ Kit (Takara). The qPCR condition was one cycle at 95 °C for 30 s, 40 cycles at 95 °C for 5 s and at 60 °C for 34 s. A melt curve was performed at the end of each reaction to verify PCR product specificity. Meanwhile, for each sample group of different stages and tissues, 300 mg insects were gathered for CDase activity assay. We collected the crude proteins of BPH in different stages and different organs to test the NlnCDase activities. The relative activities of NlnCDase enzyme were measured to confirm its hydrolytic abilities on ceramide by method mentioned above. The substrates C_12_- ceramide were excessive for the hydrolysis reactions. All qPCR reactions and CDase assay were performed independently in triplicate duplication.

### Stress treatment

To investigate how *NlnCDase* gene respond to abiotic stresses including physiological condition, diet, rice varieties and insecticides, newly emerged macropterous female adult BPH were used. For starvation treatment, newly emerged macropterous female adults were confined in a glass container with 20-day old rice seedlings or tap water as food, separately collected samples after 12, 24, 36, 48, 60, 72 h. Newly emerged macropterous female adults were separated and reared at low (22 °C), suitable (27 °C) and high (32 °C) temperatures for 7 days to investigate the thermal stimulation to the *NlnCDase* gene and its activity. Anesthetized by freezing at −20 °C for 30 s, the 3^rd^-instar nymphs were treated with 0.2 μl of three insecticides for 12 h, 24 h, 36 h or 48 h. Three insecticides, i.e. deltamethrin, imidacloprid, and triazophos, which respectively represent three types of insecticides belonging to pyrethroid, neonicotine and organic phosphorous groups, were dropped onto the thoracic terga and applied by microtopical method with acetone as control^61^. Sub-lethal concentration of the insecticides (LC_20_) (0.5ng/ul) were used for each insecticide treatment. At each time point, 10 surviving insects were collected for RNA extraction and CDase activity. Six rice varieties were used in our study, including three resistant strains (II You 7954, IR 64, Tai Ping Xian) and three susceptible strains (TN1, Xiushui 11, Shanyou 63) to feed BPH nymphs from 1^st^- to 4^th^-instar, then the 4^th^-instar nymphs were collected for further experiments. The mRNA levels were determined using the methods mentioned above.

### RNA interference of *NlnCDase*

A 457-bp (*NlnCDase*) fragment was amplified using BPH cDNA as template by PCR with forward (5′-T7-GGACCAAGTGCCAAACAAGT-3′) and reverse primers (5′-T7-GCACAGGTGGGATCAGAGAT-3′) containing the T7 primer sequence (5′-TAATACGACTCACTATAGGGAGA-3′) at the 5′ ends. The amplification reactions protocol comprised denaturing at 94 °C for 3 min, 35 cycles of denaturing at 94 °C for 30 s, annealing at 55 °C for 30 s and extension at 72 °C for 35 s, with a final extension step of 72 °C for 10 min. The fragment was transfected into PMD18 vector (Takara, China) and the sequence was verified by sequencing (Boshang company, Hangzhou, China). Meanwhile a 414-bp fragment of GFP gene (ABE28520) was amplified as a control dsRNA with forward(5′-T7-AAGTTCAGCGTGTCCGGCGA) and reverse primers (5′-T7-CACCTTGATGCCGTTCTTCT). The PCR templates for the generation of double-stranded RNA (dsRNA) were prepared as previous amplification reactions and the dsRNAs were synthsized according to the manuscript of MEGAscript ® KitHigh Yield Transcription Kit (Invitrogen). The dsRNAs were delivered into the 5th instar BPH by micro-injection and the applied dose of dsRNA were designated as 0.1 μg per insect. Five insects were collected at the 1^st^, 2^nd^ and 3^rd^ day after dsRNA injection and the transcript of *NlnCDase* was measured by qPCR to check the efficiency of RNA interference (RNAi).

### The bioassay of BPH after *NlnCDase-*dsRNA injection

To analyze the influence of *NlCDase* genes on *N*. *lugens*’s tolerance after exposing to unfriendly conditions, including starvation, high and low temperatures, we recorded the survival rate of the BPH after the dsRNA injection. All insects were pre-reared 1 day before eclosion and the newly emerged female adults were separately collected for further experiments. For the measurement of starvation adaption, the female adults were transferred into jars (10 cm deep, 5.5 cm in diameter) and reared by tap water instead of rice seedlings. The survived individuals were recorded at 12, 24, 36, 48, 60 and 72 h after the treatment. For thermal tolerance, the female adults were reared on TN1 rice seedlings at high (32 °C)and low (22 °C) temperature and the survived individuals were recorded at 1^st^, 3^rd^, 5^th^ and 7^th^ day after the treatment. About 25 insects for each sample and three samples were conducted for each treatment.

### Statistical analysis

The qPCR results were conducted according to the normalized relative quantification 2^−△△Ct^ method. Data Processing System (DPS) 13.5 was used for statistical analysis^62^. Results were represented by three independent experiments. The data were expressed as means ± S.E. Comparisons were performed by the unpaired Student’s *t*-test. One-way Analysis of Variance (ANOVA) test was used to analyze the significance among multi-groups. *P* values of <0.05 were considered statistically significant. The Least Significant Difference (LSD) method of multiple comparisons with parental and control group was applied when the probability for ANOVA was statistically significant.

## Electronic supplementary material

Supplementary file
