# Supplementary material for: A neutral ceramidase, NlnCDase, is involved in the stress responses of brown planthopper, Nilaparvata lugens (Stål)
Source: Sci Rep. 2018 Jan 18;8:1130. doi: 10.1038/s41598-018-19219-y (PMC5773612; doi:10.1038/s41598-018-19219-y)
Supplement: Supplementary file 1 — Supplementary file [file 41598_2018_19219_MOESM1_ESM.docx]

**A neutral ceramidase, *NlnCDase*, is involved in the stress responses of brown planthopper,** ***Nilaparvata lugens* (Stål)**

Xiao-Xiao SHI^1^, Yuan-Jie HUANG^1^, Mahafuj Ara BEGUM^1^, Mu-Fei Zhu^1^, Fei-Qiang LI^1^, Ming-Jing ZHANG^1^, Wen-Wu ZHOU^1*^, Cungui MAO^2^, Zeng-Rong ZHU^1*^

***Affiliations:***^1^State Key Laboratory of Rice Biology; Key Laboratory of Molecular Biology of Crop Pathogens and Insects, Ministry of Agriculture; and Institute of Insect Sciences, Zhejiang University, Hangzhou, Zhejiang, 310058, China; ^2^ Department of Medicine and Stony Brook Cancer Center, The State University of New York at Stony Brook, Stony Brook, New York 11794, USA.

***Corresponding author:****Zeng-Rong Zhu, Ph.D., Tel./ fax: +86 571 88982355. Email: [zrzhu@zju.edu.cn](mailto:zrzhu@zju.edu.cn) or Wen-Wu Zhou, Ph.D., Tel.: +86 571 88982430. Email: joewenwu@163.com.

| Exon number | Position in the cDNA | Length(bp) | Sequences at exon-intron junction |
| --- | --- | --- | --- |
| 1 | -17-141 | 158 | ttttcTAGGT-CTTTT**gt**gag |
| 2 | 142-285 | 144 | tgc**ag**ATGGG-AAGCT**gt**gag |
| 3 | 286-462 | 177 | tcc**ag**GTTCT-CCCTG**gt**cag |
| 4 | 463-590 | 128 | cgc**ag**AGCAT-GCCAA**gt**aag |
| 5 | 591-789 | 199 | ttc**ag**ATACA-GAAAG**gt**gag |
| 6 | 790-914 | 125 | ttc**ag**GGTGA-CCAGGcgccc |
| 7 | 915-999 | 85 | cccccCGACG-CCTGG**gt**gag |
| 8 | 1000-1119 | 120 | gtc**ag**GATGT-GAATG**gt**aat |
| 9 | 1120-1314 | 195 | aac**ag**GTGGA-GAGAG**gt**cag |
| 10 | 1315-1539 | 225 | ttc**ag**ATGAA-ATCAA**gt**cag |
| 11 | 1540-1635 | 96 | ttc**ag**TTGCA-AACAG**gt**acg |
| 12 | 1636-1803 | 168 | cac**ag**AAAGA-CTTTC**gt**aag |
| 13 | 1804-1916 | 113 | tgc**ag**GTGTC-ACCAA**gt**gag |
| 14 | 1917-2085 | 169 | gaa**ag**GTTCT-AGGTCagaca |
| 15 | 2086-2576 | 491 | tac**ag**GTCTT-ATCAAttttg |

**Supplementary Table S1.** Exon/Intro boundaries of the brown planthopper neutral ceramidase gene. The +1 indicates the first base of the initiation Met of the neutral CDase. Uppercase letters represent exon sequences, and lowercase letters indicate intron sequences. Boldface letters represent the intron sequences adjoining the splice junctions.


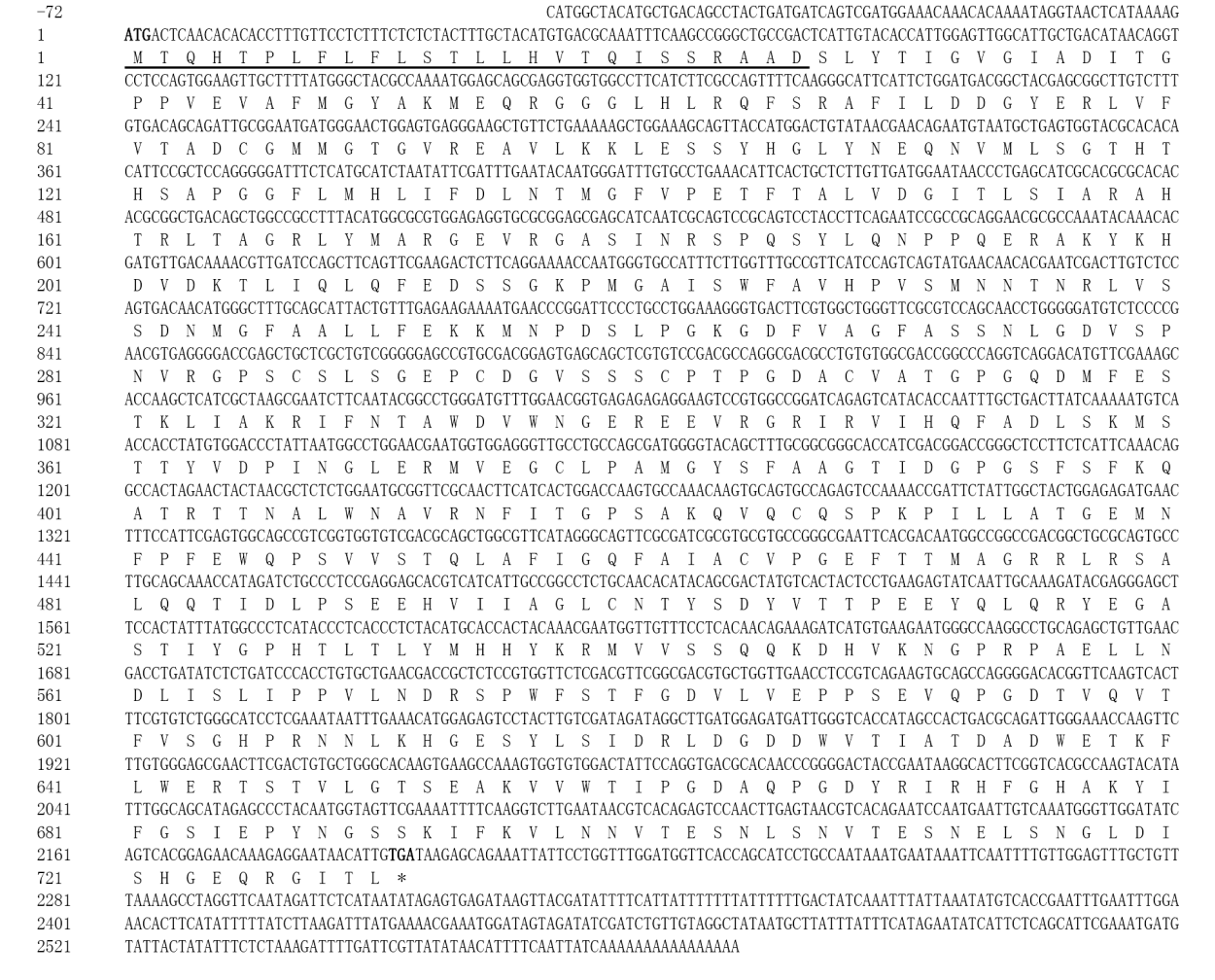


**Supplementary Figure S1.** Nucleotide and protein sequences of the NlnCDase.

The deduced amino acid sequence of NlnCDase is shown in one-letter symbols below the nucleotide sequence. The start and stop codons are boldfaced. Numbers correspond to nucleotides (*upper*) and amino acids (*lower*) at the left side. The 25 amino acids N-terminal signal sequence of the protein is underlined.


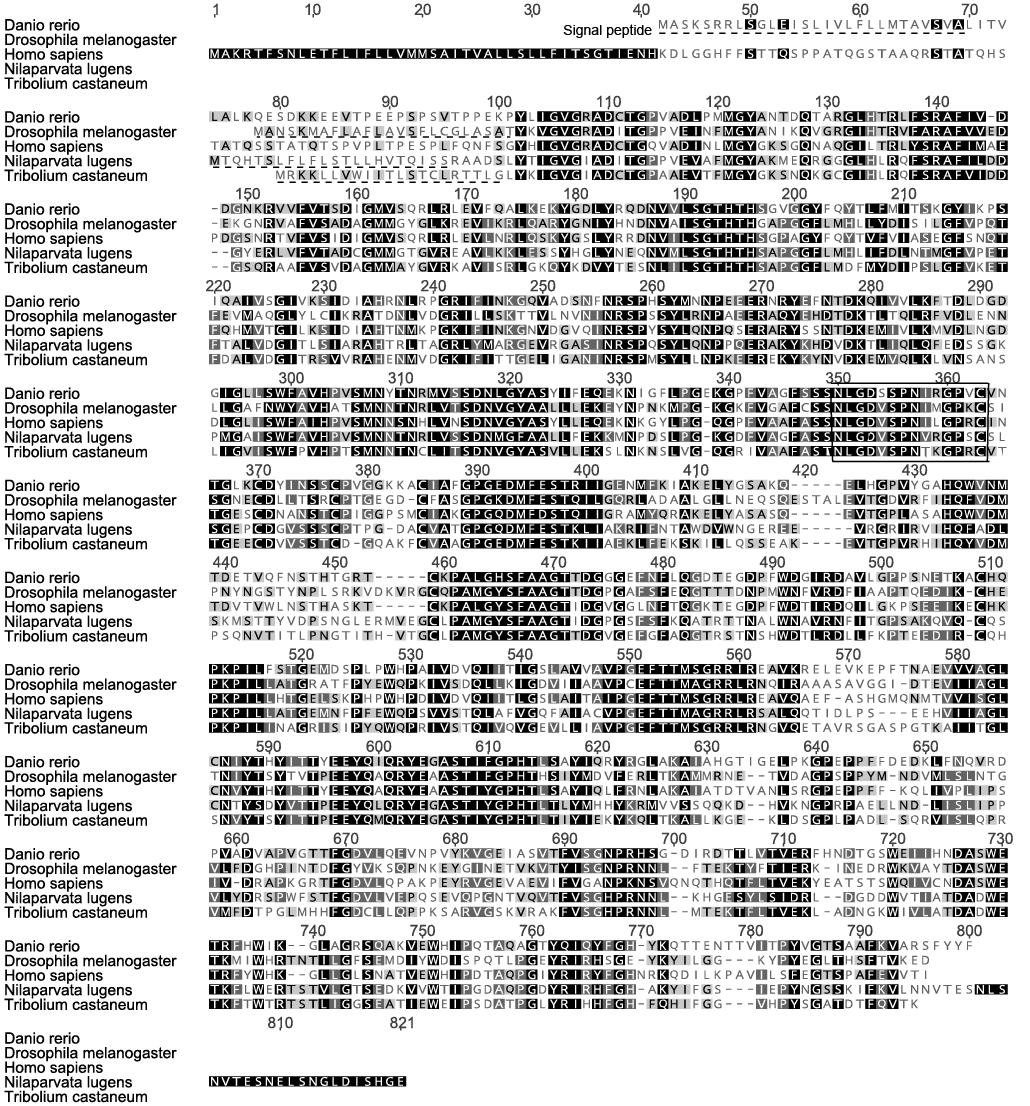


**Supplementary Figure S2.** Homology alignment of deduced amino acid sequences of neutral ceramidase from different species. The nCDases from *Danio rerio* (BAD69590), *Drosophila melanogaster* (BAC77635), *Homo sapiens* (NP_063946), *Nilaparvata lugens*, *Tribolium castaneum* (XP_968874) respectively were compared. Alignment was performed using CLUSTALW algorithm. Identical residues are shaded dark grey, and similar residues are shaded grey. The conserved ceramidase domain (NXGDVSPNXXG^P^/_X_XC) is boxed.


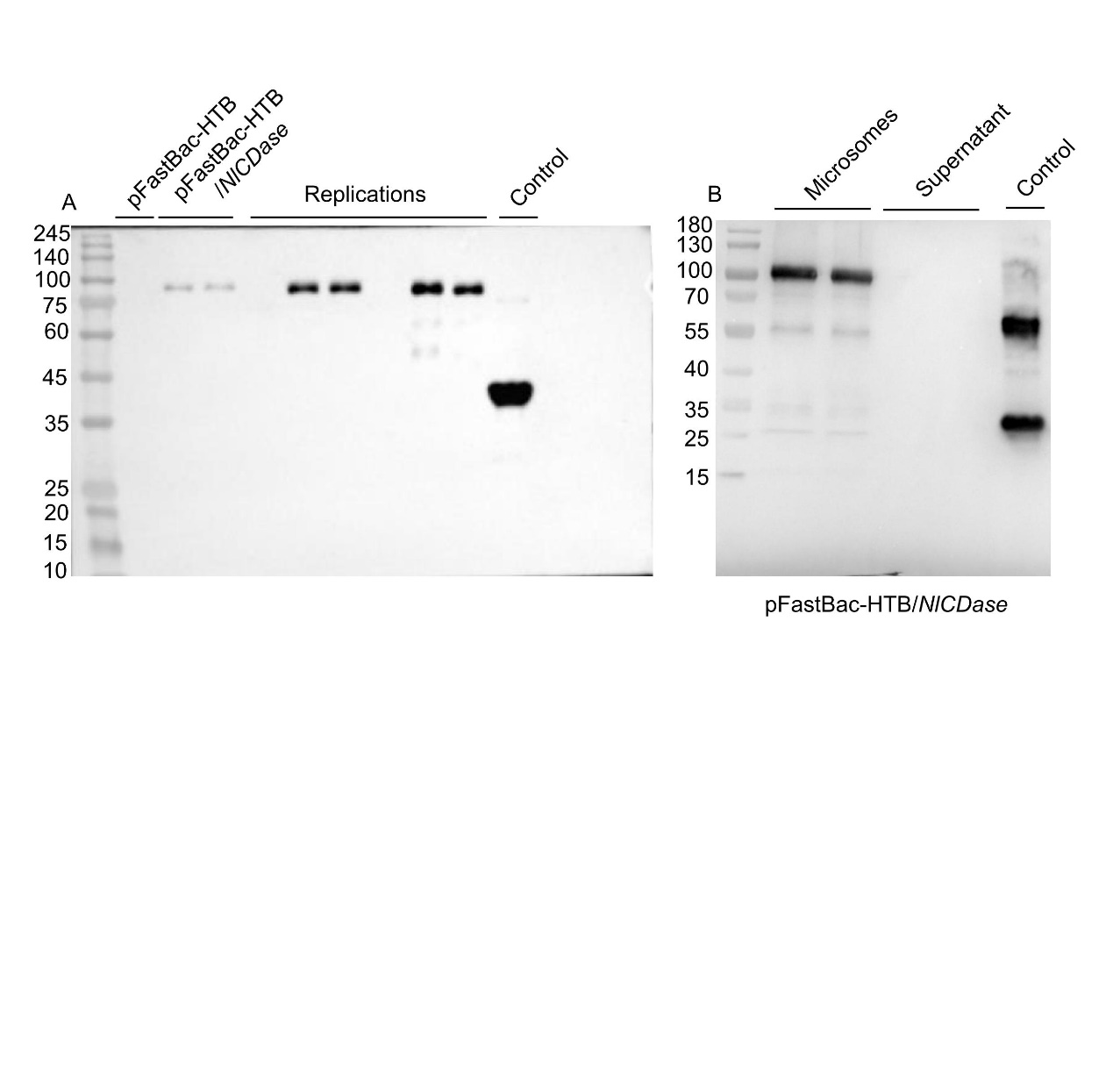


**Supplementary Figure S3.** Western Blot analysis. (A) Western Blot analysis of pFastBac-HTB and pFastBac-HTB/*NlnCDase*. (B) Western Blot analysis of microsomes and supernatant from pFastBac-HTB/*NlnCDase*.


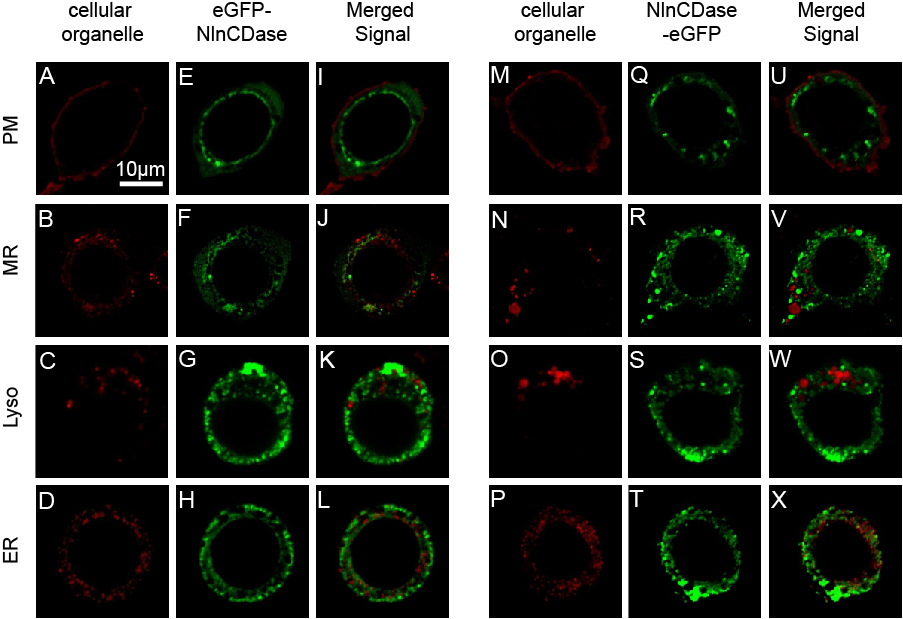


**Supplementary Figure S4.** Subcellular location of NlnCDase in High Five cells. Plasma membrane (PM) stained with DiI (A, M), mitochondria(MR) stained with Mito-Tracker (B, N), lysosome (Lyso)stained with Lyso-Tracker Red (C, O), and endoplasmic reticulum (ER)stained with ER-Tracker Red (D, P).All those organelles were in red fluorescence while the NlnCDase-eGFP (E, F, G, H) and eGFP-NlnCDase (Q, R, S, T)fusion proteins in green under the observation of confocal microscope.


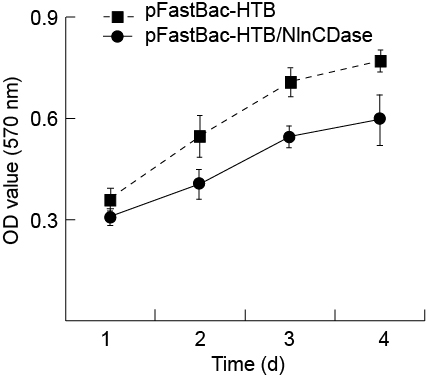


**Supplementary Figure S5.** The MTT assay of NlnCDase *in vitro*. Effect of over-expressed NlnCDase on the proliferation of High Five cells. The OD values have liner relationship with cell numbers. All data represent the mean value± SE of three independent experiments performed in duplicate.
